# Transforaminal epidural blood patch for intractable spontaneous cerebrospinal fluid leak: a case report

**DOI:** 10.1186/s40981-016-0073-2

**Published:** 2017-01-05

**Authors:** Aki Fujiwara, Keisuke Watanabe, Keiji Hashizume, Kozue Shinohara, Michiko Fukumoto, Katsuhiro Kimoto, Masahiko Kawaguchi

**Affiliations:** 1 Department of Anesthesiology, Nara Medical University, 840 Shijocho, Kashihara, Nara 634-8522 Japan; 2 Department of Pain Center, Nara Medical University, 840 Shijocho, Kashihara, Nara 634-8522 Japan; 3 Department of Pain Center, Kousei-kai Takai Hospital, 461-2 Kuranoshocho, Tenri, Nara 632-0006 Japan

**Keywords:** Transforaminal epidural blood patch, Spontaneous cerebrospinal fluid leak, Dynamic myelography

## Abstract

**Background:**

Epidural blood patch (EBP) is a recognized treatment for spontaneous cerebrospinal fluid leak (SCFL) and is typically administered by the interlaminar approach. Here, we report a case of a patient in whom SCFL failed to resolve after three applications of interlaminar EBPs before finally being successfully treated with transforaminal EBP.

**Case presentation:**

We report a case of a 41-year-old female with a definitive diagnosis of SCFL according to computed tomography (CT) myelography. A fluoroscopy-guided interlaminar EBP was applied three times without resolution of her orthostatic headache. A second myelography was therefore performed demonstrating a leak point on the ventral side of the dura mater. To close the ruptured ventral dura mater, it was necessary to fill the ventral epidural space with blood. Therefore, transforaminal EBP was performed. On spinal CT performed immediately after treatment, the ventral epidural space was observed to be filled with injected blood. Her headache improved the following day, and her symptoms completely subsided after 5 days.

**Conclusion:**

Transforaminal epidural blood patch is appropriate for patients with intractable cerebrospinal fluid leak. Patients with cerebrospinal fluid leakage due to rupture of the ventral side of the dura mater may be particularly good candidates for this procedure.

## Background

Epidural blood patch (EBP) is a recognized treatment for spontaneous cerebrospinal fluid leak (SCFL). EBP is typically performed from an interlaminar approach; however, interlaminar EBP (ILEBP) is occasionally ineffective as blood does not spread sufficiently to the leakage site. Therefore, transforaminal EBP (TFEBP) is considered an adequate alternate method for dispersing blood to the ventral epidural space in such cases. Transforaminal epidural steroid injection is a common treatment for lumbosacral radiculopathy; however, there are only few reports of transforaminal EBP. In relation to SCFL, there was only one case report which performed TFEBP after failed ILEBP [1].

Herein, we present the case of a patient with SCFL who did not respond well to ILEBPs but was subsequently treated successfully with TFEBP.

## Case presentation

We present the case of a 41-year-old female with a history of migraine and asthma. She had initially consulted a local physician for the sudden development of orthostatic headache. No abnormalities were noted on head computed tomography (CT); however, plain brain magnetic resonance imaging (MRI) revealed diffuse pachymeningeal thickening. The patient subsequently attended our hospital 23 days after symptom onset.

Orthostatic headache, hearing disorder, blurred vision, nausea, and vomiting were observed on initial examination. The patient was immediately admitted and underwent CT myelography. Retention of contrast medium was observed in the epidural space from level T2 to T8. Spontaneous cerebrospinal fluid leak (SCFL) was diagnosed accordingly. However, the leakage site could not be determined.

On the next day, fluoroscopy-guided ILEBP was performed by infusing 12 mL of a 4:1 mixture of blood and contrast medium (iohexol 240 mg/mL) at the T2/T3 level with a 22G nerve block needle (sharp needle, Hakko). On spinal CT immediately after EBP, the infused fluid was observed dispersed through the epidural space from C7 to T10. But at the lower level of thoracic epidural space, the infused blood spread insufficiently (did not cover the entire epidural space).

The patient was rested in a prone position for 3 h and then in a supine position until the next morning. As orthostatic headache did not resolve, the effect of EBP was considered insufficient.

The second ILEBP was performed 1 week after the initial procedure. A total volume of 10 mL was infused at the level T9/T10 to fill the epidural space of lower thoracic spine level. Spinal CT immediately after EBP revealed the dispersal of the administered fluid through levels C6 to T8 in the ventral epidural space and circumferentially through levels T9 to L1 in the epidural space. The way of rest after the procedure was the same as the initial procedure. This treatment was also ineffective in treating the orthostatic headache.

The third ILEBP was performed 5 days after the second procedure at the T10/T11 level. High resistance of the epidural space against infusion was noted. During ILEBP, the patient complained of back pain, and the treatment was discontinued after the infusion of 7 mL of blood. Spinal CT showed circumferential spread of the infused blood over the T10/T11 level in the epidural space. The way of rest after the procedure was the same as the previous procedures. Although the orthostatic headache was slightly alleviated on the day after the third ILEBP, symptoms recurred 2 days after the treatment.

At this time, CT images after EBP suggested that the injected blood could cover the entire epidural space where CSF leak would exist, but her symptoms did not resolve.

We thought another pathology might cause her headache. Contrast-enhanced brain MRI was performed 8 days after the third ILEBP for close evaluation of refractory headache; diffuse pachymeningeal thickening remained present with the appearance of subdural hematomas bilaterally and consequent midline shift. These findings indicated that cerebrospinal fluid (CSF) leakage persisted.

**Fig. 1:**
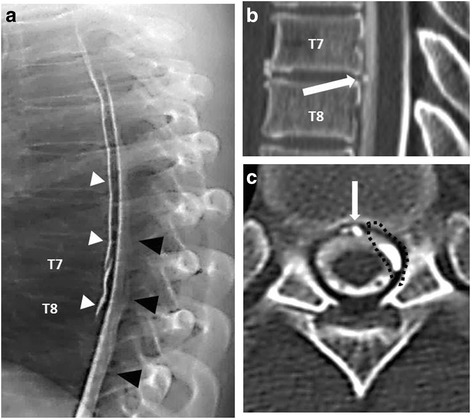
**a** Lateral dynamic myelographic image demonstrating the site of CSF leakage at T7/T8 (*white arrow*), with contrast medium observed spreading within epidural space (*white arrow heads*) from subarachnoid space (*black arrowheads*). **b**. Lateral CT myelographic image at T7/T8 demonstrating a partially calcified disc (*white arrow*). **c**. Axial CT myelographic image at T7/8 level demonstrating a collection of ventral epidural fluid (*black dotted circle*) and a partially calcified disc (*white arrow*)

On the day after contrast-enhanced MRI, CT myelography was performed again to more accurately identify the site of the CSF leakage. Dynamic myelography demonstrated the leakage of contrast medium into the ventral epidural space from the subarachnoid space at the T7/T8 intervertebral level (Fig. 1a). By reviewing images from both CT and dynamic myelography, we suspected that the sharply protruding calcified intervertebral disc had ruptured the ventral side of the dura mater, allowing for CSF to leak through the tear into the ventral epidural space (Fig. 1b, c).

**Fig. 2:**
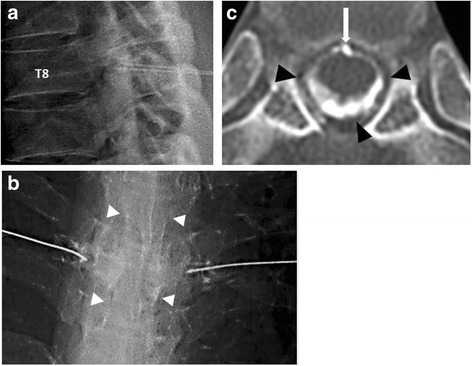
**a** Fluoroscopy-guided transforaminal EBP through bilateral T8 nerve roots (lateral view. **b** After contrast medium injection to confirm the epidural space (*white arrowheads*) (anteroposterior view. **c** Post-TFEBP spinal CT image at T7/T8 disc level demonstrating extension of injected blood through the entire epidural space (*black arrowheads*).The *white arrow* shows a partially calcified disc

On the day after the second CT myelography, fluoroscopy-guided TFEBP was performed. The patient lay on a fluoroscopy table in a prone position. After disinfection of the dorsal region, a 25G 60-mm Cathelin needle (sharp needle, Hakko) was inserted 4 cm lateral to the T8 spinous process. First of all, the needle reached the base of the transverse process of the vertebra and then passed under the transverse process of the vertebra. The needle moved forward carefully avoiding puncture of the nerve root under radiographic lateral view. After the needle tip had passed through the nerve root foramen and reached the epidural space, 1 mL of contrast medium was injected and the epidural space was confirmed under anteroposterior view. Blood and contrast medium were mixed at a ratio of 4:1, with 5 mL of this mixture injected. Diffusion was confirmed by real-time fluoroscopic imaging (Fig. 2a, b). Spinal CT immediately after this treatment showed circumferential spread of the contrast medium in the epidural space over the T7/T8 nerve root level (Fig. 2c). The patient was rested in a prone position for 3 h and then in a supine position until the next morning the same as previous procedures.

Orthostatic headache was alleviated after discontinuing rest the next morning and did not recur over the following 5 days after TFEBP, even when the patient remained seated for 2 h. The patient was discharged 1 week after TFEBP. Orthostatic headache was reported to have completely resolved 1 month after discharge. Contrast-enhanced brain MRI showed shrinking of the subdural hematomas and regression of dural thickening. No recurrence was observed 6 months after discharge.

### Discussion

CT myelography is the most sensitive method for diagnosing SCFL [2]. We definitively diagnose SCFL at our institution by performing CT myelography and confirming pooling of contrast medium in the epidural space. However, the site of dural tear is often difficult to determine.

To reduce adverse events of EBP (for example, mass effect for spinal cord or nerve roots), we infused a small amount of blood near the dural tear in cases where the tear is located. In case where the dural tear cannot be located, we infused blood over the entire pool of contrast medium in the epidural space at CT myelography image. We performed spinal CT after EBP to examine the spread of infused blood. If the spread was insufficient, we would repeat EBPs at another vertebral level with a small amount of blood [3].

In the present case, the site of CSF leakage could not be determined on initial CT myelography. Retention of contrast medium was observed in the large epidural space (from levels T2 to T8). Therefore, ILEBP was performed three times at different intervertebral levels to cover the entire area of retention of the contrast medium.

CT performed immediately after EBP confirmed that injected blood had reached the ventral epidural space. However, no marked effect was obtained, and persistent CSF leakage was observed on subsequent myelography.

Recently, the utility of digital subtraction myelography and dynamic CT myelography in identifying the site of CSF leakage has been reported [4, 5]. In the present case, the site of leakage was determined using dynamic CT myelography. As the calcified intervertebral disc was observed protruding sharply toward the ventral side of the dura mater, we assumed that the dura mater had been ruptured at this level, allowing for CSF leakage. To date, there have been a few reports of CSF leakage caused by osteophytes or herniated intervertebral discs [5–9].

The therapeutic efficacy of EBP is considered to be attributable to the volume effect of the infused blood alleviating epidural space adhesions resulting from aseptic inflammation [10–12]. In the present case, ILEBP was considered ineffective as the patching effect of infused blood was insufficient due to dilution of the infused blood by a large amount of leaked CSF. Furthermore, high resistance and pain were noted during the third ILEBP, indicating marked adhesion of the dorsal epidural space due to previous ILEBPs. In this state, the blood volume reaching the leakage site in the ventral epidural space may be insufficient with subsequent ILEBP. We therefore considered a different administration route to be necessary. In addition due to the increased risk of neuropathy as a result of increased volume effect caused by dura mater adhesions and influx of blood into the subarachnoid space under excessive infusion pressure, we judged that infusion into the dorsal epidural space should be avoided [13–17]. Eventually, although ILEBP was ineffective even though it was performed multiple times, TFEBP was found to be effective.

To our knowledge, there have been only five previous case reports of TFEBP [1, 18–21]. TFEBP was performed for post-dural puncture headache in three patients and for CSF hypovolemia in two patients. One of two case reports with CSF hypovolemia had technical difficulties with ILEBP because of precedent laminectomy. In the other case report, TFEBP was performed after failure of ILEBP as in our patient. We examined spread of injected blood after EBP by CT. It was a different point from previous case reports. TFEBP was effective in both patients.

We believe that there are three major advantages of TFEBP. First, the risk of spinal cord injury involves direct needle injury and the mass effect of epidural hematomas is lower in transforaminal approach than interlaminar approach. Second, blood can be infused near the site of CSF leakage in the ventral epidural space or near the nerve root sheath. Third, blood can be injected even in patients in whom the dorsal epidural space may be adhered (after laminectomy or multiple ILEBPs).

There are some potential complications of TFEBP. To avoid nerve root puncture, when our patient felt paresthesia during the procedure, the needle tip was withdrawn slowly and repositioned. Subarachnoid injection and intravenous injection are also risky complications of EBP. Therefore, TFEBP should be done under real-time fluoroscopy to avoid these complications. We injected contrast medium to confirm neither intrathecal injection nor intravenous injection before blood injection. The disadvantages of TFEBP include the possibility of nerve root compression due to the flow of infused blood around the nerve root and radiculopathy due to adhesion.

Further, the most appropriate infusion volume and the long-term outcomes of this procedure, including complications, remain unclear and require further evaluation.

We believe the findings of the present case demonstrate that TFEBP is a feasible alternative treatment method for patients with SCFL who respond poorly to ILEBP. Patients with CSF leakage due to rupture of the ventral side of the dura mater may be particularly good candidates for this procedure.

## Conclusions

We presented a case of a patient with spontaneous SCFL with poor response to ILEBP who was subsequently treated successfully with TFEBP. TFEBP may have utility in patients with CSF leakage due to rupture of the ventral side of the dura mater.

## Abbreviations

CSF: Cerebrospinal fluid
CT: Computed tomography
EBP: Epidural blood patch
ILEBP: Interlaminar EBP
MRI: Magnetic resonance imaging
SCFL: Spontaneous cerebrospinal fluid leak
TFEBP: Transforaminal EBP
